# New Insights into the Volume Isotope Effect of Ice Ih from Polarizable Many-Body Potentials

**DOI:** 10.1021/acs.jpclett.2c03212

**Published:** 2022-12-15

**Authors:** Soroush Rasti, Elvar Örn Jónsson, Hannes Jónsson, Jörg Meyer

**Affiliations:** † Leiden Institute of Chemistry, Gorlaeus Laboratories, Leiden University, P.O. Box 9502, 2300 RALeiden, The Netherlands; ‡ Science Institute and Faculty of Physical Sciences, University of Iceland, VR-III, 107Reykjavík, Iceland

## Abstract

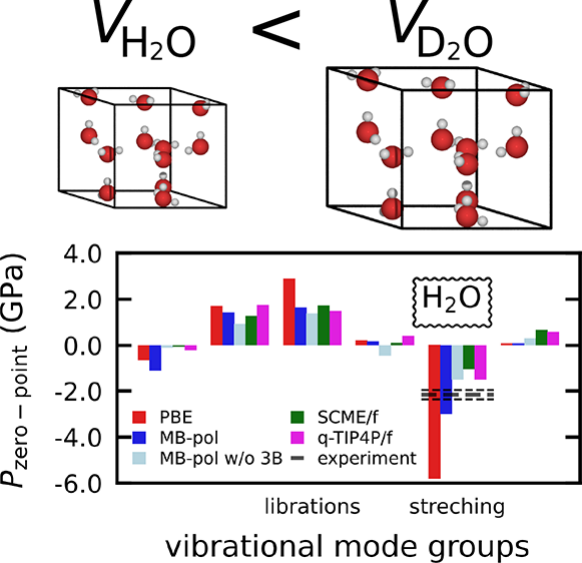

The anomalous volume isotope effect (VIE) of ice Ih is calculated and analyzed based on the quasi-harmonic approximation to account for nuclear quantum effects in the Helmholtz free energy. While a lot of recently developed polarizable many-body potential functions give a normal VIE contrary to experimental results, we find that one of them, MB-pol, yields the anomalous VIE in good agreement with the most recent high-resolution neutron diffraction measurements—better than DFT calculations. The short-range three-body terms in the MB-pol function, which are fitted to CCSD(T) calculations, are found to have a surprisingly large influence. A vibrational mode group decomposition of the zero-point pressure together with a hitherto unconsidered benchmark value for the intramolecular stretching modes of H_2_O ice Ih obtained from Raman spectroscopy data unveils the reason for the VIE: a delicate competition between the latter and the librations.

Nuclear quantum effects can manifest themselves quite prominently in macroscopic thermodynamic properties, like for example phase transition enthalpies,^1^ negative thermal expansion (NTE), or density change at low temperatures upon substitution of a light by a heavier isotope.^2^ The latter is called the volume isotope effect (VIE) and originates from the zero-point energy of the lattice vibrations (phonons). Most materials show a normal VIE, which means substitution with heavier isotopes results in a smaller molar volume at temperatures approaching the absolute zero. A hand-waving rationalization in a classical picture is that the larger vibrational amplitude and concomitant volume ascribed to a lighter isotope compared to a heavier isotope, which both experience the same chemical interaction potential at the same temperature. In ice Ih, the most common form of solid water on earth, the VIE is anomalous, resulting in a smaller unit cell and thus molar volume of the H_2_O compared to the D_2_O isotopologue (about 0.1% up to 200 K^3,4^). Despite its small magnitude, the effect has been very well quantified experimentally. Only recently, high-resolution neutron powder diffraction measurements^4^ have reduced the uncertainties for the unit cell volumes of ice Ih compared to earlier work^3^ over a wide temperature range and thus provide an excellent benchmark for atomistic interaction models that can be employed in computational studies.

Computational modeling of the VIE is very challenging. So far, water force fields ranging from simple fixed point charge up to sophisticated polarizable models have all predicted a normal VIE for ice Ih.^5−7^ Density functional theory, on the other hand, is able to model the VIE of different ice phases.^7−9^ However, even a qualitatively correct description depends very strongly on the computational settings, in particular the choice of the exchange-correlation functional.^7^ Quantification of the VIE using embedded-fragment ab initio second-order many-body perturbation (MP2) theory has fared somewhat better.^10^ But also here, the results are very sensitive to computational details like the basis set size and the embedding field. This also holds for the individual contributions of the different groups of phonon modes to the zero-point pressure, which are ultimately responsible for the VIE.^8,10^ These contributions are commonly expressed in the form of mode-specific Grüneisen parameters and have not been benchmarked against experimental data. Consequently, a detailed understanding of the VIE’s origin in terms of the competition of different contributions to the chemical interaction potential has been elusive so far.

In this work, we provide that understanding based on recently developed polarizable many-body potentials as interaction models. Building on our recent studies,^11,12^ we employ the quasi-harmonic approximation to account for nuclear quantum effects in the Helmholtz free energy by means of extensive and high-precision phonon calculations. We find that the MB-pol interaction model, whose short-range part is rooted in coupled-cluster calculations, yields the anomalous VIE of ice Ih in better agreement with the experimental reference value than DFT calculations with the PBE functional. A decomposition of the zero-point pressure into contributions from different vibrational mode groups together with a hitherto unconsidered benchmark value, which we obtain from Raman spectroscopy,^13^ allows us to scrutinize this further. According to the MB-pol total energy partitioning, the delicate competition between the librational and intramolecular stretching modes driven by a surprisingly large influence of short-range three-body effects is responsible for the anomalous VIE of ice Ih.

For the quantification of the VIE, this work employs the quasi-harmonic approximation (QHA), which has been used successfully for the same purpose in the past.^7−10,14^ According to the QHA, the Helmholtz free energy of the ice crystal is given by
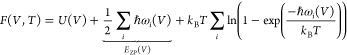
1and is conveniently evaluated per molecule. *U* is the internal energy that describes the interaction between molecules in the crystal. ω_*i*_ are the vibrational modes which determine the second (zero-point energy *E*_ZP_) and temperature-dependent third term of *F*. For the sake of simplicity, they are denoted by a collective index *i* that stands for both wave vector and band indices of the corresponding phonon modes. The internal energy *U* and the vibrational modes ω_*i*_ depend on the unit cell volume *V*, so that the minimum of *F* with respect to *V* at a given temperature is generally different for H_2_O and D_2_O isotopologues. The corresponding volumes are labeled  and  in the following. *V*_0_ minimizes *U*(*V*), with the zero reference of the latter defined such that the lattice energy *E*_lat_ = *U*(*V*_0_).

In this work, calculations with different interaction models are performed, employing and extending the Atomic Simulation Environment (ASE)^15^ for interfacing their respective implementations. This includes the fixed-point-charge-based force fields q-TIP4P/F^16,17^ (as available in LAMMPS^18^), the polarizable force fields AMOEBA14^19,20^ (as implemented in TINKER^21^), SCME/f,^12,22^ and MB-pol (as implemented in the MBX package).^23−25^ All-electron density functional theory (DFT) calculations with the PBE exchange-correlation functional^26^ are performed with the FHI-aims code,^27,28^ using the same high-accuracy settings thoroughly verified^29^ and employed^11,30^ for ice Ih in previous work (see the Supporting Information for details). The DFT calculations mimic proton disorder with a simulation cell containing 12 molecules.^31^ A simulation box containing 96 molecules, or some larger supercell, have been used for the force field interaction models to ensure the same level of convergence for *V*_0_ (±0.01 Å^3^ per molecule^29^). For all interaction models, *V*_0_ is calculated with ASE as in our earlier work by a combined optimization of the cell vectors and the molecular degrees of freedom preserving the space group of the lattice (as defined by the oxygen atoms)^11^ with a maximum force threshold of 1.0 × 10^–3^eV Å^–1^.

A continuous representation of *U*(*V*) is obtained by least-squares fitting to the Rose–Vinet^32^ equation of state. Isotropic contraction and expansion of *V*_0_ by ±4% yields 11 structures for each interaction model, for which again all molecular degrees of freedoms have been relaxed. Phonon calculations have been performed for all of these structures with the PHONOPY code,^33^ using a finite displacement^34^ of 0.02 Å in 3 × 3 × 3 supercells of the original simulation cell. The Brillouin zone has been sampled by 30 × 30 × 30 and 10 × 10 × 10 grids of phonon wave vectors in the 12 molecule and 96 molecule simulation cells, respectively. The implementation of the QHA in PHONOPY then yields a continuous representation of the volume-dependent second and third terms in eq 1 and thus , , ,^35^ and the phonon mode-dependent Grüneisen parameters γ_*i*_. Convergence checks for the VIE can be found in the Supporting Information.

Table 1 compiles experimental data and results from calculations for *V*_0_, , , and the VIE for ice Ih. A positive (negative) value corresponds to an anomalous (normal) VIE. The most recent results from the high-resolution neutron diffraction measurements by Fortes^4^ yield an even smaller and more accurately determined anomalous VIE (0.050(2)%) than the earlier data from the work of Röttger et al.^3^ (0.090(15)%). In both studies, the lowest temperature at which measurements have been performed is 10 K. For that reason, we have calculated  and  at both 0 and 10 K to confirm that this has no effect on any of the numbers presented in Table 1. As demonstrated only recently, errors related to the treatment of core and valence electrons in different DFT codes can be sizable for the calculation of energy–volume curves *U*(*V*)^36^ and could thus significantly affect results for the VIE. This is the most likely reason for the difference of 0.07% between earlier DFT results obtained with the same exchange-correlation functional (PBE).^7,14^ Our own PBE calculations eliminate this source of error and perfectly reproduce the value for *V*_0_ obtained in earlier work.^29,30^ In combination with our meticulous convergence tests for the phonon calculations with respect to the VIE (see the Supporting Information), we can therefore confirm without any further doubts the conclusions from earlier work,^7,14^ namely that the PBE functional reproduces the experimentally observed anomalous VIE but overestimates it. Likewise, our computational setup also confirms that the q-TIP4P/F force field, which is based on fixed point charges, yields a normal VIE for ice Ih.^7^ The same holds for the two polarizable force fields AMOEBA14 and the recently established SCME/f. The results for  and also for  obtained with SCME/f show the best agreement with the experimental values. SCME/f is followed by MB-pol, which also yields  and consequently a correct description of the anomalous VIE. The absolute value of 0.14% is even in much better agreement with the experimental data than (our) PBE results. This remarkable result also provides the opportunity to better understand what contributions to the chemical bonding in the ice Ih crystal are responsible for the VIE. Among all the force fields considered here, MB-pol is the only one that explicitly accounts for short-range interactions involving triples of water molecules, which have been parametrized to quantum-chemical CCSD(T) calculations.^23^ Indeed, omitting these terms (MB-pol w/o 3B in Table 1) yields a normal VIE.

**Table 1:** Volumes *V*_0_, *V*_H_2_O_, and *V*_D_2_O_ for Ice Ih^a^

	*V*_0_	*V*_H_2_O_	*V*_D_2_O_	VIE
experiments				
Fortes^4^		32.06	32.07	+0.05
Röttger et al.^3^		32.05	32.08	+0.09
calculations: DFT with PBE functional^b^				
this work	30.78	31.03	31.14	+0.36
Pamuk et al.^7^ c	29.98	30.09	30.19	+0.33
Murray and Galli^14^	30.50	30.57	30.67	+0.40
calculations: polarizable force fields				
MB-pol	31.07	31.44	31.49	+0.14
MB-pol w/o 3B	29.14	30.18	30.06	–0.39
SCME/f	30.38	31.98	31.68	–0.90
AMOEBA14	31.82	33.35	33.12	–0.67
calculations: fixed-charge force field				
q-TIP4P/F^d^	31.24	32.83	32.63	–0.61

a The volume isotope effect is quantified by VIE = *V*_D_2_O_/*V*_H_2_O_ – 1 (in percent, calculated using more decimals). The experimental data for *V*_H_2_O_, *V*_D_2_O_, and VIE have been measured at *T* = 10 K.^3,4^ Calculations from this work with all interaction models at 0 and 10 K do not yield any differences in the second decimal.

b See the Supporting Information for a collection of other values for *V*_0_ obtained with the PBE exchange-correlation functional in previous work.

c Value obtained with the QHA and a *k*-mesh of 729 points, which is most comparable to the present work.

d Note that these values differ slightly from the results obtained by Pamuk et al.^7^ due to differences in the computational setups.

At first glance, the strong influence of these terms is surprising because they do not constitute a large contribution to the cohesive (lattice) energy according to the decomposition of the internal energy at the equilibrium volume *U*(*V*_0_) in the MB-pol force field.^38^ This does not change when moving away from *V*_0_ as shown in Figure 1. Compression of the ice Ih lattice leads to an increase of the repulsive short-range interactions between pairs of water molecules (two-body terms), which is almost compensated by the increase of the long-range electrostatic and dispersion contributions. The opposite holds when expanding the volume. The one-body (i.e., deformation of individual water molecules) and short-range three-body terms play hardly any role.

**Figure 1:**
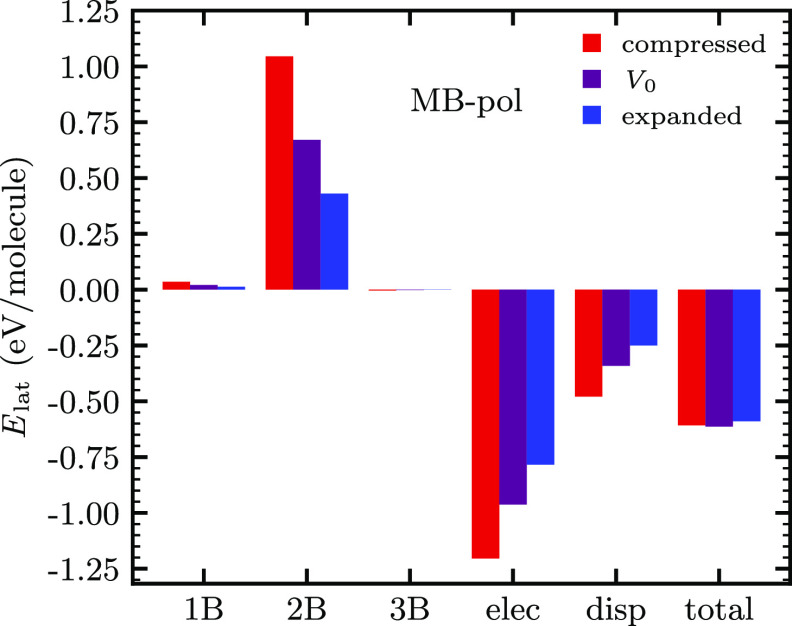
Lattice energy *E*_lat_ and its contributions according to the total energy decomposition of the MB-pol force field^23,24,37^ (all in eV per molecule). The intramolecular (1B) as well as intermolecular short-range two-body (2B), short-range three-body (3B), long-range electrostatic (elec), and dispersion (disp) contributions add up to *E*_lat_ (total). Violet, red, and blue bars depict equilibrium (*V*_0_) and isotropically compressed (0.96*V*_0_) and expanded (1.04*V*_0_) lattice configurations, respectively, as encountered during the phonon calculations for the determination of the VIE according to the QHA.

To investigate the importance of the aforementioned short-range three-body contributions (in MB-pol) for the VIE more closely, it is instructive to evaluate  and  over a wider range of temperatures, which has recently been remeasured with higher accuracy by Fortes^4^ as well. This is illustrated in the form of the relative volume changes  and  up to *T* ≤ 200 K in Figures 2a and 2b, respectively. The experimental data for H_2_O and D_2_O ice Ih shows a negative slope for *T* ≤ 60 K. This negative thermal expansion (NTE) has been modeled successfully before^10,39^ and is also reproduced by all methods considered here, except for SCME/f. This leads to a small offset in the relative volume change for H_2_O between SCME/f and the experimental data at higher temperatures, which remains almost constant. Apart from that, SCME/f captures the shape of the experimental curve for  very well, i.e., better than any other method considered here (except for MB-pol w/o 3B). MB-pol yields a slightly worse description of  similar to PBE. For , on the other hand, it provides by far the best possible description of the relative volume change (followed by PBE). Consequently, MB-pol also provides the best possible description of the VIE over the entire temperature range considered here. Not including the short-range three-body effects in MB-pol improves the shape of  but significantly worsens results for VIE(*T*), resulting in the prediction of a normal volume isotope effect. Both SCME/f and q-TIP4P/f yield an even worse shape for  compared to the experimental data. q-TIP4P/f describes a too strong NTE over a too large temperature interval (*T* ≤ 100 K) for both H_2_O and D_2_O; error canceling ultimately results in a better description of VIE(*T*) compared to SCME/f.

**Figure 2:**
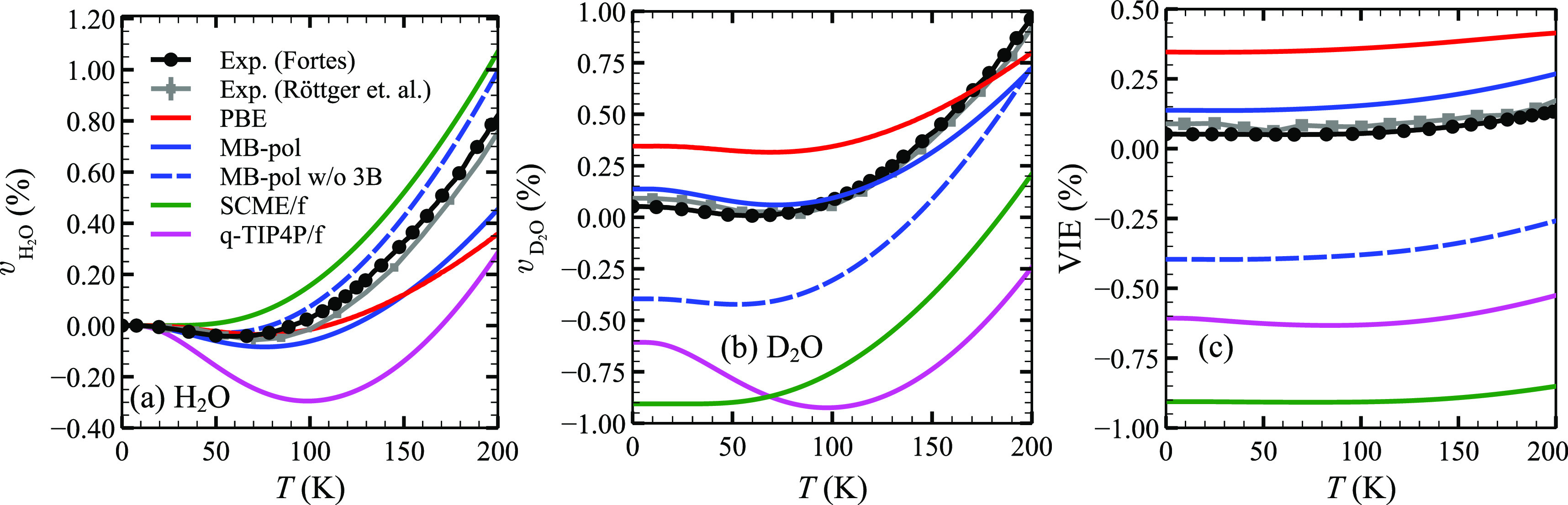
Relative volume changes (a)  and (b)  of ice Ih, both with respect to  from Table 1 for the same interaction models except AMOEBA14. (c) Resulting temperature dependence of the volume isotope effect . Error indicators for the experimental data from Röttger et al.^3^ (gray) are hardly visible, and even less so for the data from Fortes^4^ (black), whereby lines are meant to guide the eye.

According to the QHA (see eq 1) the temperature dependence of the equilibrium volume is completely determined by the vibrational modes. As demonstrated in previous work,^5,7,8,10^ their change upon compression and expansion can be analyzed in detail by means of the mode-dependent Grüneisen parameters , which define the zero-point pressure
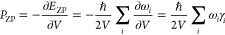
2Positive (negative) values for *P*_ZP_ yield expansion (contraction) of the volume due to zero-point energy effects. Figures 3a and 3b show that all methods yield a positive total zero-point pressure for both H_2_O or D_2_O, respectively, as to be expected according to the results compiled in Table 1. For an anomalous (normal) VIE (at 0 K), the total *P*_ZP_ needs to be smaller (larger) for H_2_O than for D_2_O. The corresponding differences are shown in Figure 3c, and indeed only PBE and MB-pol yield . Figure 3 also shows a decomposition of the zero-point pressure into contributions from the five different vibrational mode groups characterized by hydrogen-bond bending (HB) and stretching (HS), librations (L), intramolecular bending (B), and stretching (S). Unlike the differences of the total *P*_ZP_, which unfortunately cannot be measured directly, the differences between the contributions from the mode groups vary much more when considering different interaction models. The *P*_ZP_ contribution from the HB and HS groups is hardly affected by H–D substitution (see Figure 3c), which is not surprising because both of these mode groups involve the frustrated translation of entire H_2_O and D_2_O molecules. Likewise, all methods suggest that the B modes contribute very little to the VIE and that it is the delicate balance between the expansive *P*_ZP_ of the L modes (frustrated rotations) and the contractive *P*_ZP_ of the S modes, which predominantly determine the sign of the zero-point pressures difference between both isotopologues.

**Figure 3:**
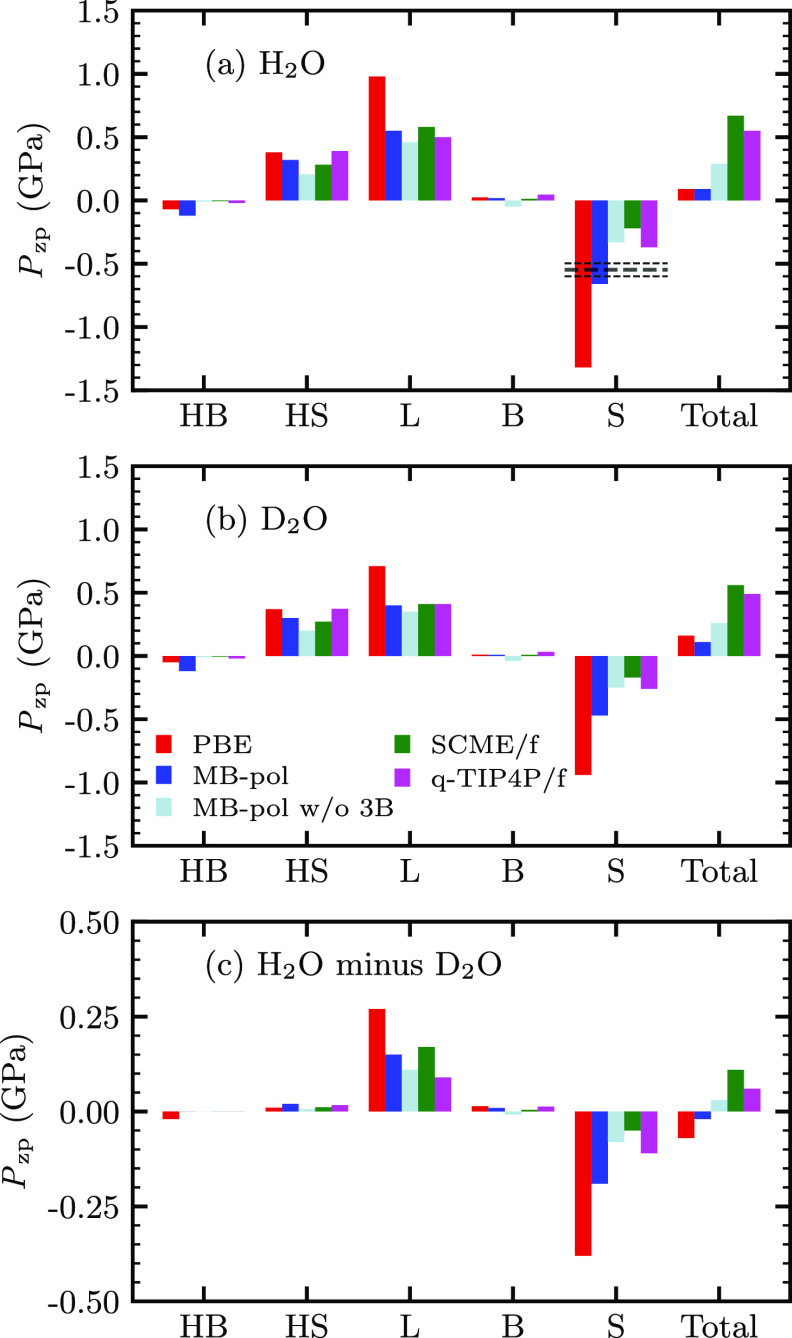
Total zero-point pressure *P*_ZP_ (rightmost bars) and its decomposition according to eq 2 into the vibrational mode groups composed of hydrogen-bond bending (HB, 0–125 cm^–1^), hydrogen-bond stretching (HS, 125–500 cm^–1^), librations (L, 500–1500 cm^–1^ (425–900 cm^–1^) in H_2_O (D_2_O) ice Ih), intramolecular bending (B, 1500–2000 cm^–1^ (900–1300 cm^–1^) in H_2_O (D_2_O) ice Ih), and intramolecular stretching (S, 3000–4000 cm^–1^ (2000–3000 cm^–1^) in H_2_O (D_2_O) ice Ih) modes in (a) H_2_O and (b) D_2_O) ice Ih. Results are shown using the same interaction models as in Figure 2. (c) Differences between (a) and (b) (*P*_ZP_(H_2_O) – *P*_ZP_(D_2_O)). An estimate for  based on experimental data for H_2_O ice Ih is indicated in (a) (see text and the Supporting Information for details).

Salim et al.^10^ have already pointed that the subtle interplay of the *P*_ZP_ contributions from different mode groups makes it additionally challenging to determine whether a particular interaction model captures the VIE correctly for the right reason. We have already noted in our earlier work^11^ that the measurements by Minceva-Sukarova et al.^13^ of the pressure dependence of the Raman peak for the S mode group in H_2_O ice Ih at 246 K play a key role in this context. As further detailed in the Supporting Information, this allows us to obtain a good estimate for the contribution by the S mode group  −0.548(51) GPa, which is based on experimental data alone. Figure 3a includes this value as a black horizontal line. Considering that error estimates are lower bounds, MB-pol almost reproduces this value exactly (−0.66 GPa) and clearly comes much closer than PBE (−1.32 GPa) and MB-pol w/o 3B (−0.33 GPa). This confirms that short-range three-body effects play indeed a very important role for the correct atomistic description of the VIE.

Unfortunately, Minceva-Sukarova et al.^13^ have not measured the S mode group frequency shift for the D_2_O isotopologue of ice Ih. Because all interaction models suggest a strongly localized character of the S modes, the outcome of such a measurement can be estimated based on the reduced masses associated with the O–H and O–D bonds as  −0.399 GPa (see the Supporting Information), which is consequently again in excellent agreement with the MB-pol value in Figure 3b (−0.47 GPa). While it would be good to see this value confirmed in future experiments, the outstanding performance of MB-pol and the concomitant understanding would be better scrutinized by experimental data for the L modes. Ideally, such data could also be measured at temperatures much lower than 246 K to avoid systematic errors related to mode softening effects in future comparisons of experiments and theory.

In summary, this study provides new insights into the volume isotope effect of ice Ih based on calculations within the quasi-harmonic approximation by employing a variety of different interaction models. Numerically precise all-electron DFT calculations with the PBE exchange-correlation functional provide a reference value and confirm that PBE yields an anomalous VIE but largely overestimates its magnitude. Among the three state-of-the-art polarizable force fields only MB-pol yields an anomalous VIE, the magnitude of which is in significantly better agreement with the most recent experimental data than PBE. A detailed analysis based on the MB-pol energy partitioning reveals a surprisingly large influence of the cooperative short-range interaction between three water molecules for the VIE and the temperature dependence of the volume of H_2_O and D_2_O ice Ih up to 200 K. Finally, the zero-point pressure is decomposed into contributions from different vibrational mode groups, and an estimate from experimental data for the contribution from the intramolecular stretching modes of H_2_O ice Ih is extracted, which is completely independent from all measurements related to the VIE. Among all interaction models considered here, this is in best agreement with MB-pol—while being significantly overestimated by PBE and underestimated by all other force fields. This suggests that MB-pol yields the anomalous VIE of ice Ih for the right reason. It thus also enables unprecedented quantification and atomic-scale understanding of its driving force, namely the delicate competition between the expansive librational and contractive intramolecular stretching modes upon substitution of hydrogen by deuterium, mitigated by short-range three-body effects. Future computational studies should investigate other ice polymorphs. However, before embarking on this endeavor, it would be highly desirable to have experimental benchmark data for contribution from other vibrational mode groups to the zero-point pressure, ideally with smaller error bars and for both H_2_O and D_2_O isotopologues. This bears the exciting prospect of obtaining fundamental insights about volume isotope effects in those other polymorphs as well and to establish a delicate benchmark that can be used for the further development and testing of interaction models targeting condensed forms of water. Altogether, this could provide a new angle for the fundamental understanding of hydrogen bonding in these most paradigmatic systems.

## Supporting Information Available

Comparison of the unit cell volumes for ice Ih and ice VIII obtained in previous DFT calculations, convergence tests for the VIE calculations, zero-point pressures from experimental data (PDF)

## References

[ref1] Fuentes-Landete V.; Rasti S.; Schlögl R.; Meyer J.; Loerting T. Calorimetric Signature of Deuterated Ice II: Turning an Endotherm to an Exotherm. J. Phys. Chem. Lett. 2020, 11, 8268–8274. 10.1021/acs.jpclett.0c02368. 32902994 PMC7528406

[ref2] Pounder E. R. The Physics of Ice; Pergamon: Oxford, 1965.

[ref3] Röttger K.; Endriss A.; Ihringer J.; Doyle S.; Kuhs W. F. Lattice Constants and Thermal Expansion of H_2_O and D_2_O Ice I *h* between 10 and 265 K. Acta Cryst. B 1994, 50, 644–648. 10.1107/S0108768194004933. 22267563

[ref4] Fortes A. D. Accurate and Precise Lattice Parameters of H_2_O and D_2_O Ice Ih between 1.6 and 270 K from High-Resolution Time-of-Flight Neutron Powder Diffraction Data. Acta Cryst. B 2018, 74, 196–216. 10.1107/S2052520618002159. 29616994

[ref5] Herrero C. P.; Ramírez R. Isotope Effects in Ice Ih: A Path-Integral Simulation. J. Chem. Phys. 2011, 134, 094510 10.1063/1.3559466. 21384988

[ref6] Ramírez R.; Neuerburg N.; Fernández-Serra M.-V.; Herrero C. P. Quasi-Harmonic Approximation of Thermodynamic Properties of Ice Ih, II, and III. J. Chem. Phys. 2012, 137, 044502 10.1063/1.4737862. 22852626

[ref7] Pamuk B.; Soler J. M.; Ramírez R.; Herrero C. P.; Stephens P. W.; Allen P. B.; Fernández-Serra M.-V. Anomalous Nuclear Quantum Effects in Ice. Phys. Rev. Lett. 2012, 108, 193003 10.1103/PhysRevLett.108.193003. 23003032

[ref8] Umemoto K.; Sugimura E.; de Gironcoli S.; Nakajima Y.; Hirose K.; Ohishi Y.; Wentzcovitch R. M. Nature of the Volume Isotope Effect in Ice. Phys. Rev. Lett. 2015, 115, 173005 10.1103/PhysRevLett.115.173005. 26551113

[ref9] Umemoto K.; Wentzcovitch R. M. First Principles Study of Volume Isotope Effects in Ices VIII and X. Jpn. J. Appl. Phys. 2017, 56, 05fa03 10.7567/JJAP.56.05FA03.

[ref10] Salim M. A.; Willow S. Y.; Hirata S. Ice Ih Anomalies: Thermal Contraction, Anomalous Volume Isotope Effect, and Pressure-Induced Amorphization. J. Chem. Phys. 2016, 144, 204503 10.1063/1.4951687. 27250312

[ref11] Rasti S.; Meyer J. Importance of Zero-Point Energy for Crystalline Ice Phases: A Comparison of Force Fields and Density Functional Theory. J. Chem. Phys. 2019, 150, 234504 10.1063/1.5097021. 31228884

[ref12] Jónsson E. O.; Rasti S.; Galynska M.; Meyer J.; Jónsson H. Transferable Potential Function for Flexible H_2_O Molecules Based on the Single Center Multipole Expansion. J. Chem. Theory Comput. 2022, 10.1021/acs.jctc.2c00598. 36395502

[ref13] Minceva-Sukarova B.; Sherman W. F.; Wilkinson G. R. The Raman Spectra of Ice (Ih, II, III, V, VI and IX) as Functions of Pressure and Temperature. J. Phys. C: Solid State Phys. 1984, 17, 5833–5850. 10.1088/0022-3719/17/32/017.

[ref14] Murray É. D.; Galli G. Dispersion Interactions and Vibrational Effects in Ice as a Function of Pressure: A First Principles Study. Phys. Rev. Lett. 2012, 108, 105502 10.1103/PhysRevLett.108.105502. 22463422

[ref15] Larsen A. H.; Mortensen J. J.; Blomqvist J.; Castelli I. E.; Christensen R.; Dułak M.; Friis J.; Groves M. N.; Hammer B.; Hargus C.; et al. The Atomic Simulation Environment — a Python Library for Working with Atoms. J. Phys.: Condens. Matter 2017, 29, 273002 10.1088/1361-648X/aa680e. 28323250

[ref16] Abascal J. L.; Vega C. A General Purpose Model for the Condensed Phases of Water: TIP4P/2005. J. Chem. Phys. 2005, 123, 234505 10.1063/1.2121687. 16392929

[ref17] Habershon S.; Markland T. E.; Manolopoulos D. E. Competing Quantum Effects in the Dynamics of a Flexible Water Model. J. Chem. Phys. 2009, 131, 024501 10.1063/1.3167790. 19603998

[ref18] Plimpton S. Fast Parallel Algorithms for Short-Range Molecular Dynamics. J. Comput. Phys. 1995, 117, 1–19. 10.1006/jcph.1995.1039.

[ref19] Ren P.; Ponder J. W. Polarizable Atomic Multipole Water Model for Molecular Mechanics Simulation. J. Phys. Chem. B 2003, 107, 5933–5947. 10.1021/jp027815+.

[ref20] Laury M. L.; Wang L.-P.; Pande V. S.; Head-Gordon T.; Ponder J. W. Revised Parameters for the AMOEBA Polarizable Atomic Multipole Water Model. J. Phys. Chem. B 2015, 119, 9423–9437. 10.1021/jp510896n. 25683601 PMC4772747

[ref21] Rackers J. A.; Wang Z.; Lu C.; Laury M. L.; Lagardère L.; Schnieders M. J.; Piquemal J.-P.; Ren P.; Ponder J. W. Tinker 8: Software Tools for Molecular Design. J. Chem. Theory Comput. 2018, 14, 5273–5289. 10.1021/acs.jctc.8b00529. 30176213 PMC6335969

[ref22] Wikfeldt K. T.; Batista E. R.; Vila F. D.; Jónsson H. A Transferable H_2_O Interaction Potential Based on a Single Center Multipole Expansion: SCME. Phys. Chem. Chem. Phys. 2013, 15, 16542–16556. 10.1039/c3cp52097h. 23949215

[ref23] Babin V.; Medders G. R.; Paesani F. Development of a “First Principles” Water Potential with Flexible Monomers. II: Trimer Potential Energy Surface, Third Virial Coefficient, and Small Clusters. J. Chem. Theory Comput. 2014, 10, 1599–1607. 10.1021/ct500079y. 26580372

[ref24] Babin V.; Leforestier C.; Paesani F. Development of a “First Principles” Water Potential with Flexible Monomers: Dimer Potential Energy Surface, VRT Spectrum, and Second Virial Coefficient. J. Chem. Theory Comput. 2013, 9, 5395–5403. 10.1021/ct400863t. 26592277

[ref25] Medders G. R.; Babin V.; Paesani F. A Critical Assessment of Two-Body and Three-Body Interactions in Water. J. Chem. Theory Comp. 2013, 9, 1103–1114. 10.1021/ct300913g. 26588754

[ref26] Perdew J. P.; Burke K.; Ernzerhof M. Generalized Gradient Approximation Made Simple. Phys. Rev. Lett. 1996, 77, 3865–3868. 10.1103/PhysRevLett.77.3865. 10062328

[ref27] Blum V.; Gehrke R.; Hanke F.; Havu P.; Havu V.; Ren X.; Reuter K.; Scheffler M. Ab Initio Molecular Simulations with Numeric Atom-Centered Orbitals. Comp. Phys. Comm. 2009, 180, 2175–2196. 10.1016/j.cpc.2009.06.022.

[ref28] Havu V.; Blum V.; Havu P.; Scheffler M. Efficient O(N) Integration for All-Electron Electronic Structure Calculation Using Numeric Basis Functions. J. Comput. Phys. 2009, 228, 8367–8379. 10.1016/j.jcp.2009.08.008.

[ref29] Santra B.; Klimeš J.; Tkatchenko A.; Alfè D.; Slater B.; Michaelides A.; Car R.; Scheffler M. On the Accuracy of van Der Waals Inclusive Density-Functional Theory Exchange-Correlation Functionals for Ice at Ambient and High Pressures. J. Chem. Phys. 2013, 139, 154702 10.1063/1.4824481. 24160528

[ref30] Sun J.; Remsing R. C.; Zhang Y.; Sun Z.; Ruzsinszky A.; Peng H.; Yang Z.; Paul A.; Waghmare U.; Wu X.; et al. Accurate First-Principles Structures and Energies of Diversely Bonded Systems from an Efficient Density Functional. Nat. Chem. 2016, 8, 831–836. 10.1038/nchem.2535. 27554409

[ref31] Hamann D. R. H_2_O Hydrogen Bonding in Density-Functional Theory. Phys. Rev. B 1997, 55, R10157–r10160. 10.1103/PhysRevB.55.R10157.

[ref32] Vinet P.; Smith J. R.; Ferrante J.; Rose J. H. Temperature Effects on the Universal Equation of State of Solids. Phys. Rev. B 1987, 35, 1945–1953. 10.1103/PhysRevB.35.1945. 9941621

[ref33] Togo A.; Tanaka I. First Principles Phonon Calculations in Materials Science. Scripta Mater. 2015, 108, 1–5. 10.1016/j.scriptamat.2015.07.021.

[ref34] Parlinski K.; Li Z. Q.; Kawazoe Y. First-Principles Determination of the Soft Mode in Cubic ZrO_2_. Phys. Rev. Lett. 1997, 78, 4063–4066. 10.1103/PhysRevLett.78.4063.

[ref35] This work uses the same convention as in the recent work of Fortes^4^ to quantify the volume isotope effect, i.e., VIE = (*V*_D_2_O_ – *V*_H_2_O_)/*V*_H_2_O_, whereas others have used VIE′ = (*V*_H_2_O_ – *V*_D_2_O_)/*V*_D_2_O_, which leads to almost the same absolute numbers but flips the sign. Both expressions are related according to VIE = −VIE′/(1 + VIE′) and VIE′ = −VIE/(1 + VIE), respectively. For the sake of brevity, the temperature dependence has been omitted.

[ref36] Lejaeghere K.; Bihlmayer G.; Bjorkman T.; Blaha P.; Blugel S.; Blum V.; Caliste D.; Castelli I. E.; Clark S. J.; Dal Corso A.; et al. Reproducibility in Density Functional Theory Calculations of Solids. Science 2016, 351, aad3000 10.1126/science.aad3000. 27013736

[ref37] Medders G. R.; Babin V.; Paesani F. Development of a “First-Principles” Water Potential with Flexible Monomers. III. Liquid Phase Properties. J. Chem. Theory Comput. 2014, 10, 2906–2910. 10.1021/ct5004115. 26588266

[ref38] Pham C. H.; Reddy S. K.; Chen K.; Knight C.; Paesani F. Many-body Interactions in Ice. J. Chem. Theory Comp. 2017, 13, 1778–1784. 10.1021/acs.jctc.6b01248. 28245359

[ref39] Gupta M. K.; Mittal R.; Singh B.; Mishra S. K.; Adroja D. T.; Fortes A. D.; Chaplot S. L. Phonons and Anomalous Thermal Expansion Behavior of H_2_O and D_2_O Ice Ih. Phys. Rev. B 2018, 98, 104301 10.1103/PhysRevB.98.104301.

