# Supplementary material for: New Insights into the Volume Isotope Effect of Ice Ih from Polarizable Many-Body Potentials
Source: J Phys Chem Lett. 2022 Dec 15;13(50):11831–6. doi: 10.1021/acs.jpclett.2c03212 (PMC9791686; doi:10.1021/acs.jpclett.2c03212)
Supplement: Supplementary file 1 — jz2c03212_si_001.pdf [file jz2c03212_si_001.pdf]

Supporting Information: New Insights into the Volume Isotope Effect of Ice Ih from Polarizable Many-Body Potentials

Soroush Rasti,[†] Elvar Örn Jónsson,[‡] Hannes Jónsson,[‡] and Jörg Meyer[*,†]

†*Leiden Institute of Chemistry, Gorlaeus Laboratories, Leiden University, P.O. Box 9502, 2300 RA Leiden, The Netherlands*

‡*Science Institute and Faculty of Physical Sciences, University of Iceland, VR-III, 107 Reykjavík, Iceland*

E-mail: j.meyer@chem.leidenuniv.nl

Contents

1 Comparison to Previous DFT Calculations S-3

2 Convergence Tests for the VIE Calculations S-4

3 Zero-Point Pressures from Experimental Data S-6

References S-8

1 Comparison to Previous DFT Calculations

Table S1: Volumes V_0 for ice Ih (see main text for definition) as obtained "directly" from DFT calculations (i.e., without considering ZPE effects) with the PBE exchange-correlation functional. Results from this work, Pamuk et al.[S1] and[S2] are already given in Table 1in the main article. Equivalent results for ice VIII are shown to further illustrate difference between all-electron calculations and those which employ approximations for core and valence electrons. All values are in Å^3 per molecule.

	V_0(ice Ih)	V_0(ice VIII)
all-electron calculations		
this work	30.78	20.73
Santra et al.[S3], Sun et al.[S4]	30.79	20.74
calculations with approximations for core and valence electrons		
Pamuk et al.[S1]	29.98	
Murray and Galli[S2]	30.50	20.44
Umemoto and Wentzcovitch[S5]		20.12
Feibelman[S6]	30.65	
Brandenburg et al.[S7]	30.15	20.36
Liu and Ojamäe[S8]	30.3	20.5

All DFT calculations in this work employ the PBE exchange-correlation functional[S9]. They have been carried out the with the all-electron DFT code FHI-aims[S10,S11], which employs numerically tabulated atom-centered orbitals as basis set. The standard tight settings and tier-2 and tier-3 basis sets for hydrogen and oxygen atoms, respectively. A $4 \times 4 \times 4$ Monkhorst-Pack grid[S12] is employed for Brillouin zone sampling. These settings are the same as those established by Santra et al.[S3], whose thorough convergence tests gave an estimate of $\pm 0.01\,\text{Å}^3$ for the numerical accuracy of V_0 with respect to these settings. Sun et al.[S4] have used the same settings afterwards, As to be expected and shown in Table S1, all three calculations agree perfectly within the aforementioned accuracy margin.

Table S1 also reveals that DFT-PBE calculations with approximations for the treatment of core and valence electrons (pseudopotentials) systematically underestimate V_0 for both ice Ih and ice VIII. A more detailed investigation of this interesting finding is outside the scope of this work.

2 Convergence Tests for the VIE Calculations

Three sets of convergence tests have been carried out, to scrutinize the accuracy of the results reported in Table 1 in the main article for V_{H_2O} and V_{D_2O} (and thus VIE) with respect to the following three parameters:

1. Accuracy of geometry optimization for the water molecules in the simulation cell. This is quantified by the force threshold F_{max} criterion that is used to stop the relaxation. Smaller values for F_{max} yield for accurate results.

2. Finite displacement in phonon calculations Δ_{disp}. For force fields where analytic formulations of forces are available, smaller values of Δ_{disp} reduce the error for the second derivatives in the phonon calculations. However, in DFT calculations, forces (usually) come with a numerical error, the reduction of which requires additional computational effort (i.e., increasing the accuracy of the self-conistent field cycle). Therefore, a small value that is as large as possible not to affect V_{H_2O} and V_{D_2O} (and thus VIE) is sought after here.

3. Range of isotropic contraction and expansion of V_0 used for the construction of a continuous representation of the volume-dependent terms required for the quasi-harmonic approximation (QHA) given by Equation 1 in the main article. Different intervals $[(1-s_{max})V_0; (1+s_{max})V_0]$ have been considered, which are all discretized by 11 equidistant points. For too small intervals the numerical noise in the volumes gains a too large influence on the fit, while too large intervals leave the regime of validity for the QHA.

For reasons of computational convenience, all calculations for these convergence tests have been carried with the AMOEBA14 force field. All the results are compile in Table S2. Accurate results can be obtained with $F_{max} = 1 \times 10^{-3}\,\text{eV}\,\text{Å}^{-1}$, $\Delta_{disp} = 0.02\,\text{Å}$ and $s_{max} = 4\,\%$. As described in the main text, this is what has been used for all interaction models throughout this work.

Table S2: Results of convergence tests with respect to the three parameters F_{max}, Δ_{disp} and s_{max} as defined in the text. Note that the volumes $V_{\mathrm{H_2O}}$ and $V_{\mathrm{D_2O}}$ (for ice Ih) are rounded to two decimals, and that VIE $= \frac{V_{\mathrm{D_2O}}}{V_{\mathrm{H_2O}}} - 1$ is calculated using more decimals.

F_{max} (eV Å^{-1})	Δ_{disp} (Å)	s_{max} (%)	$V_{\mathrm{H_2O}}$ (Å^3/H_2O)	$V_{\mathrm{D_2O}}$ (Å^3/D_2O)	VIE (%)
Force threshold criterion for geometry optimizations					
1.0×10^{-4}	0.02	4.0	33.35	33.12	−0.67
1.0×10^{-3}	0.02	4.0	33.35	33.12	−0.67
2.5×10^{-3}	0.02	4.0	33.36	33.12	−0.69
5.0×10^{-3}	0.02	4.0	33.37	33.13	−0.69
Finite displacement for phonon calculations					
1.0×10^{-3}	0.01	4.0	33.35	33.12	−0.67
1.0×10^{-3}	0.02	4.0	33.35	33.12	−0.67
1.0×10^{-3}	0.03	4.0	33.36	33.12	−0.68
1.0×10^{-3}	0.06	4.0	33.37	33.13	−0.68
1.0×10^{-3}	0.08	4.0	33.38	33.14	−0.69
Maximum expansion and contraction of cell volume for the QHA					
1.0×10^{-3}	0.02	0.2	33.23	33.02	−0.60
1.0×10^{-3}	0.02	0.5	33.21	33.02	−0.56
1.0×10^{-3}	0.02	1.0	33.26	33.07	−0.54
1.0×10^{-3}	0.02	2.0	33.29	33.10	−0.60
1.0×10^{-3}	0.02	3.0	33.33	33.11	−0.68
1.0×10^{-3}	0.02	4.0	33.35	33.12	−0.67
1.0×10^{-3}	0.02	5.0	33.35	33.12	−0.67

3 Zero-Point Pressures from Experimental Data

Using Raman spectroscopy Minceva-Sukarova et al.[S13] measured the shift of the intramolecular stretching mode peak in the H_2O-isotopologue of ice Ih at 246 K when applying external pressure

$$\frac{\partial \nu_{\mathrm{S}}}{\partial P} = (-78.0 \pm 7.2)\,\mathrm{cm}^{-1}\,\mathrm{GPa}^{-1} \quad , \tag{1}$$

which yields

$$h\frac{\partial \nu_{\mathrm{S}}}{\partial P} = (1.549 \pm 0.143) \times 10^{-21}\,\mathrm{J\,GPa}^{-1} \tag{2}$$

($1\,h\,\mathrm{cm}^{-1} = 1.9863 \times 10^{-23}\,\mathrm{J}$). This allows us to obtain an estimate based on experimental data for the zero-point pressure of the intramolecular stretching mode group

$$P_{\mathrm{zp}}^{\mathrm{S}} \approx -\sum_{i\in \mathrm{S}} \frac{\hbar}{2}\frac{\partial \omega_i}{\partial V} = \frac{1}{2}\frac{B_0}{V_0}\,h\sum_{i\in \mathrm{S}}\frac{\partial \nu_i}{\partial P} \quad , \tag{3}$$

where $\frac{\partial \omega}{\partial V} = \frac{\partial P}{\partial V}\frac{\partial \omega}{\partial P} = -2\pi\frac{B_0}{V_0}\frac{\partial \nu}{\partial P}$. In the following, it is assumed that $\frac{\partial \nu_{\mathrm{S}}}{\partial P}$ does not change significantly with temperature.

The highly accurate experimental values for the bulk modulus $B_0 = 11.33\,\mathrm{GPa}$ (extrapolated to 0 K)[S14] and the unit cell volume $V_0 = 32.05\,\mathrm{\AA}^3$ per H_2O molecule[S15] (at 10 K), result in

$$\frac{1}{2}\frac{B_0}{V_0} = 0.177\,\mathrm{GPa\,\AA}^{-3} \quad . \tag{4}$$

Assuming the same shift for both symmetric and antisymmetric stretching modes and neglecting dispersion ($\frac{\partial \nu_i}{\partial P} = \frac{\partial \nu_{\mathrm{S}}}{\partial P}$), which is consistent with the Raman experiments of Minceva-Sukarova et al.[S13][1], result in

$$h\sum_{i\in \mathrm{S}}\frac{\partial \nu_i}{\partial P} \approx 2\cdot h\frac{\partial \nu_{\mathrm{S}}}{\partial P} \approx -(3.099 \pm 0.286) \times 10^{-21}\,\mathrm{J\,GPa}^{-1} \tag{5}$$

where the factor of two comes from the summation over both stretching modes per molecule.

[1] These experiments only yield results for the Γ-point.

This leads to the final value

$$P_{\mathrm{zp}}^{\mathrm{S}} \approx (-0.548 \pm 0.051)\,\mathrm{GPa} \tag{6}$$

($1\,\mathrm{J}\,\mathrm{\AA}^{-3} = 1 \times 10^{30}\,\mathrm{Pa}$). Due to aforementioned approximations, the indicated errors should be considered as lower bounds.

Unfortunately, Minceva-Sukarova et al.[S13] do not report $\frac{\partial \nu_{\mathrm{S}}}{\partial P}$ for D_2O ice Ih, and to the best of our knowledge no such measurement is available. Despite the relatively strong hydrogen bonding in ice Ih[S16], the S modes are still largely dominated by the local intramolecular potential along a single O−H bond. This leads to a simple one-dimensional picture, where the relative shift of the corresponding O−D stretching mode is (approximately) given by

$$\alpha = \frac{\nu_{\mathrm{S}}^{\mathrm{D_2O}}}{\nu_{\mathrm{S}}^{\mathrm{H_2O}}} \approx \frac{\mu_{\mathrm{O-H}}}{\mu_{\mathrm{O-D}}} = \sqrt{\frac{m_{\mathrm{H}}}{m_{\mathrm{D}}}\frac{m_{\mathrm{O}}+m_{\mathrm{D}}}{m_{\mathrm{O}}+m_{\mathrm{H}}}} \approx 0.728 \quad , \tag{7}$$

where $\mu_{\mathrm{O-X}} = \frac{m_{\mathrm{X}} \cdot m_{\mathrm{O}}}{m_{\mathrm{X}}+m_{\mathrm{O}}}$ ($\mathrm{X} \in \{\mathrm{H}, \mathrm{D}\}$) is the reduced mass of the corresponding O−X bond with $m_{\mathrm{H}} = 1.0078\,\mathrm{u}$, $m_{\mathrm{D}} = 2.0141\,\mathrm{u}$, $m_{\mathrm{O}} = 15.999\,\mathrm{u}$. The calculations with all interaction models considered in this work leads to values between 0.726 and 0.730 for α, which confirms that deviations from this one-dimensional picture are very small. Consequently, measurements should yield

$$\frac{\partial \nu_{\mathrm{S}}^{\mathrm{D_2O}}}{\partial P} \approx \frac{\partial(\alpha \nu_{\mathrm{S}}^{\mathrm{H_2O}})}{\partial P} \approx 0.728 \cdot \frac{\partial \nu_{\mathrm{S}}^{\mathrm{H_2O}}}{\partial P} \tag{8}$$

for the shift of the intramolecular stretching mode peak in the D_2O-isotopologue of ice Ih.

In fact, Minceva-Sukarova et al.[S13] do provide the average stretching mode for both H_2O ($\nu_{\mathrm{OH(H_2O)}} = 3138\,\mathrm{cm}^{-1}$) and D_2O ($\nu_{\mathrm{OD(D_2O)}} = 2316\,\mathrm{cm}^{-1}$) ice Ih at 246 K, which – without considering uncertainties – yields $\alpha = 0.738$.

References

(S1) Pamuk, B.; Soler, J. M.; Ramírez, R.; Herrero, C. P.; Stephens, P. W.; Allen, P. B.; Fernández-Serra, M.-V. Anomalous Nuclear Quantum Effects in Ice. *Phys. Rev. Lett.* **2012**, *108*, 193003, DOI: 10.1103/PhysRevLett.108.193003.

(S2) Murray, É. D.; Galli, G. Dispersion Interactions and Vibrational Effects in Ice as a Function of Pressure: A First Principles Study. *Phys. Rev. Lett.* **2012**, *108*, 105502, DOI: 10.1103/PhysRevLett.108.105502.

(S3) Santra, B.; Klimeš, J.; Tkatchenko, A.; Alfè, D.; Slater, B.; Michaelides, A.; Car, R.; Scheffler, M. On the Accuracy of van Der Waals Inclusive Density-Functional Theory Exchange-Correlation Functionals for Ice at Ambient and High Pressures. *J. Chem. Phys.* **2013**, *139*, 154702, DOI: 10.1063/1.4824481.

(S4) Sun, J.; Remsing, R. C.; Zhang, Y.; Sun, Z.; Ruzsinszky, A.; Peng, H.; Yang, Z.; Paul, A.; Waghmare, U.; Wu, X.; Klein, M. L.; Perdew, J. P. Accurate First-Principles Structures and Energies of Diversely Bonded Systems from an Efficient Density Functional. *Nat. Chem.* **2016**, *8*, 831–836, DOI: 10.1038/nchem.2535.

(S5) Umemoto, K.; Wentzcovitch, R. M. Amorphization in Quenched Ice VIII: A First-Principles Study. *Phys. Rev. B* **2004**, *69*, 180103, DOI: 0.1103/PhysRevB.69.180103.

(S6) Feibelman, P. J. Lattice Match in Density Functional Calculations: Uce Ih vs. β-AgI. *Phys. Chem. Chem. Phys.* **2008**, *10*, 4688–4691, DOI: 10.1039/b808482n.

(S7) Brandenburg, J. G.; Maas, T.; Grimme, S. Benchmarking DFT and Semiempirical Methods on Structures and Lattice Energies for Ten Ice Polymorphs. *J. Chem. Phys.* **2015**, *142*, 124104, DOI: 10.1063/1.4916070.

(S8) Liu, Y.; Ojamäe, L. Raman and IR Spectra of Ice Ih and Ice XI with an Assessment

of DFT Methods. *J. Phys. Chem. B* **2016**, *120*, 11043–11051, DOI: 10.1021/acs.jpcb.6b07001.

(S9) Perdew, J. P.; Burke, K.; Ernzerhof, M. Generalized Gradient Approximation Made Simple. *Phys. Rev. Lett.* **1996**, *77*, 3865–3868, DOI: 10.1103/PhysRevLett.77.3865.

(S10) Blum, V.; Gehrke, R.; Hanke, F.; Havu, P.; Havu, V.; Ren, X.; Reuter, K.; Scheffler, M. Ab Initio Molecular Simulations with Numeric Atom-Centered Orbitals. *Comp. Phys. Comm.* **2009**, *180*, 2175–2196, DOI: 10.1016/j.cpc.2009.06.022.

(S11) Havu, V.; Blum, V.; Havu, P.; Scheffler, M. Efficient O(N) Integration for All-Electron Electronic Structure Calculation Using Numeric Basis Functions. *J. Comput. Phys.* **2009**, *228*, 8367–8379, DOI: 10.1016/j.jcp.2009.08.008.

(S12) Monkhorst, H. J.; Pack, J. D. Special Points for Brillouin-Zone Integrations. *Phys. Rev. B* **1976**, *13*, 5188–5192, DOI: 10.1103/PhysRevB.13.5188.

(S13) Minceva-Sukarova, B.; Sherman, W. F.; Wilkinson, G. R. The Raman Spectra of Ice (Ih, II, III, V, VI and IX) as Functions of Pressure and Temperature. *J. Phys. C: Solid State Phys.* **1984**, *17*, 5833–5850, DOI: 10.1088/0022-3719/17/32/017.

(S14) Neumeier, J. J. Elastic Constants, Bulk Modulus, and Compressibility of H_2O Ice Ih for the Temperature Range 50 K –273 K. *J. Phys. Chem. Ref. Data* **2018**, *47*, 033101, DOI: 10.1063/1.5030640.

(S15) Fortes, A. D. Accurate and Precise Lattice Parameters of H_2O and D_2O Ice Ih between 1.6 and 270 K from High-Resolution Time-of-Flight Neutron Powder Diffraction Data. *Acta Cryst. B* **2018**, *74*, 196–216, DOI: 10.1107/s2052520618002159.

(S16) Santra, B.; Klimeš, J.; Alfè, D.; Tkatchenko, A.; Slater, B.; Michaelides, A.; Car, R.; Scheffler, M. Hydrogen Bonds and van Der Waals Forces in Ice at Ambient and High

Pressures. *Phys. Rev. Lett.* **2011**, *107*, 185701, DOI: 10.1103/PhysRevLett.107.185701.
